# Supplementary material for: A metabolically stable PET tracer for imaging synaptic vesicle protein 2A: synthesis and preclinical characterization of [18F]SDM-16
Source: Eur J Nucl Med Mol Imaging. 2021 Nov 11;49(5):1482–96. doi: 10.1007/s00259-021-05597-5 (PMC8940841; doi:10.1007/s00259-021-05597-5)
Supplement: Supplementary file 1 — Supplementary file1 (DOCX 448 KB) [file 259_2021_5597_MOESM1_ESM.docx]

**A metabolically stable PET probe for imaging the synaptic vesicle protein 2A: Synthesis and preclinical characterization of [^18^F]SDM-16**

Chao Zheng^1^, Daniel Holden^1^, Ming-Qiang Zheng^1^, Richard Pracitto^1^, Kyle C. Wilcox^2^, Marcel Lindemann^1^, Zachary Felchner^1^, Li Zhang^1^, Jie Tong^1^, Krista Fowles^1^, Sjoerd J. Finnema^2^, Nabeel Nabulsi^1^, Richard E. Carson^1^, Yiyun Huang^1^, Zhengxin Cai^1^

1. PET Center, Department of Radiology and Biomedical Imaging, Yale School of Medicine, New Haven, CT 06520, USA
2. Translational Imaging Neuroscience, AbbVie, North Chicago, IL 60064, USA

**Methods**

**Chemistry and general conditons**. All reagents and solvents were purchased from commercial sources (Sigma-Aldrich, VWR, and Fisher Scientific, etc.) and used without further purification. Proton, carbon, and fluorine nuclear magnetic resonance (^1^H, ^13^C, and ^19^F NMR) spectra were recorded on an Agilent 400 or 500 MHz spectrometer. Chemical shifts are reported in parts per million (ppm), with either the solvent resonance or tetramethyl silane (TMS) as the internal standard (TMS, 0 ppm; CDCl_3_, 7.26 ppm; DMSO-*d*_6_, 2.49 ppm in ^1^H NMR and CDCl_3_, 77.0 ppm; DMSO-*d*_6_, 39.7 ppm in ^13^C NMR). Multiplicities are indicated as s (singlet), d (doublet), t (triplet), q (quartet), quint (quintet), m (multiplet); coupling constants *J* are given in Hertz (Hz). All chemicals used in this study were of ≥ 95% purity based on HPLC (detection at 254 nm), or NMR. HRMS (ESI) data were tested on a Shimadzu 9030 QToF LC-MS system.

**Chiral Separation of 4-(3,5-difluorophenyl)pyrrolidin-2-one (9)**. Compound **9** was synthesized in 45% yield over 3 steps according to the previously reported procedure [1]. The separation was conducted on a ChiralPak AS-H preparative HPLC column (250 x 30 mm I.D., 5µm), eluting with *n*-heptane/EtOH (3 : 2 v/v), with the active enantiomer (*R*)-**9** eluting first, followed by the inactive enantiomer (*S*)-**9**. The *e.e.* was determined on an analytical Chiralpak IA column (5 μm, 4.6 x 150 mm) eluting with *n*-hexane/EtOH (1 : 1 v/v) at a flow rate of 1.00 mL/min. Both enantiomers showed >99% ee. (*R*)-**9**: R_t_ = 5.81 min, (*S*)-**9**: R_t_ = 6.25 min. ^1^H NMR (CDCl_3_, 400 MHz): *δ* 6.78 (d, *J* = 6.44 Hz, 2H), 6.77-6.63 (m, 1H), 6.61 (s, 1H), 3.78 (t, *J* = 10.0 Hz, 1H), 3.65 (q, *J* = 16.0 Hz, 1H), 3.38 (t, *J* = 8.0 Hz, 1H), 2.74 (dd, *J* = 16.0, 8.0 Hz, 1H), 2.42 (dd, *J* = 16.0, 8.0 Hz, 1H). ^13^C NMR (CDCl_3_, 100 MHz): *δ* 37.5, 39.8, 48.9, 102.6 (t, *J* = 25.0 Hz), 109.7 (dd, *J* = 19.0, 7.0 Hz, 2C), 146.0 (t, *J* = 9.0 Hz), 163.2 (dd, *J* = 248.0, 13.0 Hz, 2C), 176.9. ^19^F NMR (CDCl_3_, 376 MHz): *δ* -108.96.

**4-(3,5-difluorophenyl)-1-(hydroxymethyl)pyrrolidin-2-one (10)**. In a 2.5 ml V-vial were placed compound **9** (0.05 g, 0.253 mmol), 37% formalin solution (38 µl, 0.507 mmol) and water (0.5 ml) mixed, and heated at 115 °C for 16 h. After cooling to room temperature, the suspension was dissolved in methanol (10 ml), the solvents were removed under reduced pressure, and the residue was purified by column chromatography (SiO_2_, 19/1/0.01, CH_2_Cl_2_/MeOH/NH_4_OH, v/v/v) to give **10** (55 mg, 96%). ^1^H NMR (CDCl_3_, 500 MHz): *δ* 2.50 (dd, *J* = 17.1, 7.7 Hz, 1H), 2.84 (dd, *J* = 17.1, 8.8 Hz, 1H), 3.55-3.61 (m, 2H), 3.93 (t, *J* = 8.2 Hz, 1H), 4.46 (s, 1H), 4.77 (d, *J* = 8.7 Hz, 1H), 4.91 (d, *J* = 9.2 Hz, 1H), 6.69 (t, *J* = 8.9 Hz, 1H), 6.77 (d, *J* = 5.9 Hz, 2H); ^13^C NMR (CDCl_3_, 125 MHz): *δ* 37.0, 39.1, 52.7, 66.5, 102.8 (t, *J* = 25.3 Hz), 109.9 (dd, *J* = 19.2, 5.8 Hz, 2C), 146.3 (t, *J* = 8.8 Hz), 163.4 (dd, *J* = 249.2, 12.9 Hz, 2C), 174.4. ^19^F NMR (CDCl_3_, 470 MHz): *δ* -108.85.

**4-(3,5-difluorophenyl)-1-((2-methyl-1*H*-imidazol-1-yl)methyl)pyrrolidin-2-one (*rac* SDM-16, 7)**. In a two-neck round bottom flask 2-methylimidazole (50 mg, 0.616 mmol), and *N*, *N*-diisopropylethylamine (100 µl, 0.572 mmol) were dissolved in acetonitrile (4.5 ml). To this mixture oxalyl chloride (22 µl, 0.264 mmol) dissolved in acetonitrile (0.5 ml) were slowly added. Under argon atmosphere the mixture was stirred for 30 min at room temperature. Then (100 mg, 0.440 mmol) ***rac* 10** were added, stirred for 30 min at room temperature and further heated under refluxe for 5.5 h. After cooling to room temperature, the solvent was removed under reduced pressure and the crude mixture was purified by column chromatography (I: SiO_2_, 19/1/0.01 CH_2_Cl_2_/MeOH/NH_4_OH, v/v/v and II: SiO_2_, gradient system 100-80% ethyl acetate/ethanol) to give **13** (59 mg, 46%). ^1^H NMR (CDCl_3_, 400 MHz): *δ* 2.43 (s, 3H), 2.51 (dd, *J* = 17.2, 8.2 Hz, 1H), 2.83 (dd, *J* = 17.2,  9.0 Hz, 1H), 3.29 (dd, *J* = 9.3, 6.9 Hz, 1H), 3.49-3.57 (m, 1H), 3.69 (t, *J* = 8.8 Hz, 1H), 5.31-5.42 (m, 2H), 6.64-6.71 (m, 3H), 6.91 (s, 2H); ^13^C NMR (CDCl_3_, 100 MHz): *δ* 12.9, 29.6, 36.8 (t, *J* = 1.8 Hz), 37.9, 52.1 (d, *J* = 3.3 Hz), 102.9 (t, *J* = 25.2 Hz), 109.7 (dd, *J* = 17.9, 7.0 Hz, 2C), 119.4, 128.1, 144.9, 145.1 (t, *J* = 8.8 Hz), 163.3 (dd, *J* = 249.7 Hz, *J* = 12.9 Hz, 2C), 173.1. ^19^F NMR (CDCl_3_, 376 MHz): *δ* -108.41. HRMS (ESI) Calcd for C_15_H_16_N_3_OF_2_^+^ [M+H]^+^: 292.1256, found 292,1280.

**(2-methyl-1*H*-imidazol-1-yl)methanol (12)**. 2-methyl-1*H*-imidazole (3.0 g, 36.54 mmol), paraformaldehyde (1.21 g, 40.19mmol), and triethylamine (0.037 g, 0.36 mmol) were mixed, heated at 100 ^o^C until 2-methyl-1*H*-imidazole disappear, about 30 min, afford colorless solid 4.07 g. After washed with Et_2_O afford **12** in quantitative yield. ^1^H NMR (DMSO-*d*_6_, 400 MHz): *δ* 2.27 (s, 3H), 5.17 (s, 2H), 6.67 (s, 1H), 7.02 (s, 1H); ^13^C NMR (DMSO-*d*_6_, 100 MHz): *δ* 12.8, 68.5, 119.9, 126.4, 144.2.

**1-(chloromethyl)-2-methyl-1*H*-imidazole hydrogen chloride (13)**. Compound **12** (4.1 g, 36.54 mmol) was dissolved in thionyl chloride (SOCl_2_) (10 ml) at 0 ^o^C, then stirred at room temperature for 3 h, the solvents were removed under reduced pressure, dried in vacuo to afforded **13** (5.5 g, 90%), mixed with 10% 2-methyl-1*H*-imidazol-1-yl)methanol hydrochloride (hydrochloride of **12**). ^1^H NMR (DMSO-*d*_6_, 400 MHz): *δ* 2.64 (s, 3H), 6.12 (s, 2H), 7.53 (d, *J* = 4.0 Hz, 1H), 7.75 (d, *J* = 4.0 Hz, 1H); ^13^C NMR (DMSO-*d*_6_, 100 MHz): *δ* 10.6, 53.3, 119.1, 122.4, 146.4.

**Chiral Separation of 4-(3-bromo-5-fluorophenyl)pyrrolidin-2-one (15)**. Compound **15** was synthesized in 60% yield over 3 steps according to the previously reported procedure [1]. The separation was conducted on a ChiralPak AS-H preparative HPLC column (250 x 30 mm I.D., 5µm), eluting with *n*-heptane/EtOH (3 : 2 v/v), with the active enantiomer (*R*)-15 eluting first, followed by the inactive enantiomer (*S*)-**15**. The *e.e.* was determined on an analytical Chiralpak IA column (5 μm, 4.6 x 150 mm) eluting with 50:50 EtOH/hexane at a flow rate of 1.00 mL/min. Both enantiomers showed >99% ee. (*R*)-**15**: R_t_ = 5.39 min, (*S*)-**15**: R_t_ = 5.73 min. ^1^H NMR (CDCl_3_, 400 MHz): *δ* 7.17-7.11 (m, 2H), 6.88 (d, *J* = 8.0 Hz, 1H), 6.55 (s, 1H), 3.77 (t, *J* = 8.0 Hz, 1H), 3.62 (q, *J* = 8.0 Hz, 1H), 3.37 (t, *J* = 8.0 Hz, 1H), 2.71 (dd, *J* = 16.0, 8.0 Hz, 1H), 2.41 (dd, *J* = 16.0, 8.0 Hz, 1H). ^13^C NMR (CDCl_3_, 100 MHz): *δ* 37.6, 39.6, 49.0, 112.8 (d, *J* = 22.0 Hz), 117.9 (d, *J* = 22.0 Hz), 123.0 (d, *J* = 10.0 Hz), 125.8 (d, *J* = 3.0 Hz), 146.2 (dd, *J* = 8.0 Hz, 3.0 Hz), 162.5 (d, *J* = 250.0 Hz), 177.0. ^19^F NMR (CDCl_3_, 376 MHz): *δ* -109.83.

**(*R*)-4-(3,5-difluorophenyl)-1-((2-methyl-1*H*-imidazol-1-yl)methyl)pyrrolidin-2-one (SDM-16, *R*-7)**. To a solution of compound **(*R*)-9** (10 mg, 0.05 mmol) in anhydrous THF (0.5 ml) under argon and cooled to 0 ^o^C was added sodium hydride (NaH, 5 mg, 0.11 mmol). Tetrabutylammonium iodide (TBAI, 1 mg, 0.003 mmol) and compound **13** (20 mg, 0.12 mmol) were added after 30 min. The reaction mixture was kept stirring for 16 h at room temperature, then quenched with saturated NaHCO_3_ solution (1 ml) and extracted with EtOAc (5 ml x 3). The combined organic phase was dried over Na_2_SO_4_ and concentrated *in vacuo*. The crude product was purified on a silica gel column eluting with 0-10% EtOH/EtOAc to afford compound (***R***)**-7** as an oil (13 mg, 92%). ^1^H NMR (CDCl_3_, 400 MHz): *δ* ^1^H NMR (CDCl_3_, 400 MHz): *δ* 2.43 (s, 3H), 2.51 (dd, *J* = 17.2 Hz, 8.2 Hz, 1H), 2.83 (dd, *J* = 17.2 Hz, 9.0 Hz, 1H), 3.29 (dd, *J* = 9.3 Hz, 6.9 Hz, 1H), 3.49-3.57 (m, 1H), 3.69 (t, *J* = 8.8 Hz, 1H), 5.31-5.42 (m, 2H), 6.64-6.71 (m, 3H), 6.91 (s, 2H); ^13^C NMR (CDCl_3_, 100 MHz): *δ* 12.9, 29.6, 36.8 (t, *J* = 1.8 Hz), 37.9, 52.1 (d, *J* = 3.3 Hz), 102.9 (t, *J* = 25.2 Hz), 109.7 (dd, *J* = 17.9, 7.0 Hz, 2C), 119.4, 128.1, 144.9, 145.1 (t, *J* = 8.8 Hz), 163.3 (dd, *J* = 249.7, 12.9 Hz, 2C), 173.1. ^19^F NMR (CDCl_3_, 376 MHz): *δ* -108.40.

**(*R*)-4-(3-bromo-5-fluorophenyl)-1-((2-methyl-1*H*-imidazol-1-yl)methyl)pyrrolidin-2-one (16)**. Compound **16** was prepared in procedures similar to those described in supporting information for ***rac* SDM-16** (**7)**. Yield 90%. ^1^H NMR (CDCl_3_, 400 MHz): *δ* 2.37 (s, 3H), 2.45 (dd, *J* = 20.0, 8.0 Hz, 1H), 2.76 (dd, *J* = 20.0, 8.0 Hz, 1H), 3.24 (t, *J* = 8.0 Hz, 1H), 3.43-3.51 (m, 1H), 3.65 (t, *J* = 10.0 Hz, 1H), 5.26-5.36 (m, 2H), 6.73 (d, *J* = 12.0 Hz, 1H), 6.85 (d, *J* = 8.0, 2H), 7.03 (s, 1H), 7.06 (d, *J* = 8.0, 1H). ^13^C NMR (CDCl_3_, 100 MHz): *δ* 12.9, 36.5, 36.5, 37.9, 52.0 (d, *J* = 5.0 Hz), 112.7 (d, *J* = 22.0 Hz), 118.1 (d, *J* = 24.0 Hz), 119.4, 123.1 (d, *J* = 10.0 Hz), 125.6 (d, *J* = 3.0 Hz), 128.0, 144.8, 145.3 (d, *J* = 8.0 Hz), 163.5 (d, *J* = 245.0 Hz), 173.0. ^19^F NMR (CDCl_3_, 376 MHz): *δ* -109.46.

**(*R*)-4-(3-fluoro-5-(trimethylstannyl)phenyl)-1-((2-methyl-1*H*-imidazol-1-yl)methyl)pyrrolidin-2-one (17).** To a solution of compound **16** (60 mg, 0.17 mmol) in anhydrous toluene (0.62 ml) was added lithium chloride (44 mg, 1.02 mmol), tetrakis(triphenylphosphine)palladium (0) (20 mg, 0.02 mmol), triphenylphosphine (2 mg, 0.01 mmol), and hexamethylditin (57 µL, 90 mg, 0.27 mmol) under argon. The reaction mixture was degassed and refilled with argon for 3 min and kept stirring at 100 ^o^C for 1 h. The reaction mixture was diluted with EtOAc (2 ml), passed through celite, and rinsed with EtOAc (2 mL x 2). The filtrate was concentrated in vacuo. The crude product was purified on a silica gel column eluting with 0-20% EtOH/EtOAc to afford the product **17** as an oil (34 mg, 46%). ^1^H NMR (CDCl_3_, 400 MHz): *δ* 0.20-0.33 (m, 9H), 2.44 (s, 3H), 2.56 (dd, *J* = 16.0 Hz, 8.0 Hz, 1H), 2.83 (dd, *J* = 16.0 Hz, 8.0 Hz, 1H), 3.30 (t, *J* = 8.0 Hz, 1H), 3.54 (m, 1H), 3.68 (t, *J* = 8.0 Hz, 1H), 5.30-5.43 (m, 2H), 6.74-6.77 (m, 1H), 6.90 (s, 2 H), 6.99 (s, 1H), 7.04-7.06 (m, 1H); ^13^C NMR (CDCl_3_, 100 MHz): *δ* -9.4 (3H), 12.9, 36.9, 38.2, 52.1, 52.6, 113.2 (d, *J* = 22.0 Hz), 119.5, 121.1 (d, *J* = 17.00 Hz), 121.5 (d, *J* = 2.00 Hz), 143.1 (d, *J* = 6.00 Hz), 144.9, 146.5, 162 (d, *J* = 251 Hz), 164.0, 173.7.

1. Li S, Cai Z, Wu X, Holden D, Pracitto R, Kapinos M, et al. Synthesis and in Vivo Evaluation of a Novel PET Radiotracer for Imaging of Synaptic Vesicle Glycoprotein 2A (SV2A) in Nonhuman Primates. ACS chemical neuroscience. 2019;10:1544-54. doi:10.1021/acschemneuro.8b00526.

**Fig S1**. Plasma over blood ratio (PoB) of [^18^F]SDM-16.

**Fig S2**. baseline scan and Levetiracetam displacement scan on same monkey

**Fig S3**. Comparation of input function with TACs.

**Table S1.** Average residence times of [^18^F]SDM-16 in selected organs.

| **Organ** | **N (h)_Female** | **N (h)_Male** |
| --- | --- | --- |
| Brain | 0.532 | 0.430 |
| Gall Bladder | 0.015 | 0.021 |
| Heart | 0.015 | 0.017 |
| Kidneys | 0.057 | 0.067 |
| Liver | 0.305 | 0.335 |
| Urinary Bladder | 0.193 | 0.143 |
| Remainder | 1.504 | 1.608 |
| Sum | 2.621 | 2.621 |

**Table S2.** Mean organ radiation dose estimates (mSv/MBq) for [^18^F]SDM-16 in adult female and male phantoms.

| **Organ** | **mGv/MBq (Female)** | **mGv/MBq (Male)** |
| --- | --- | --- |
| Adrenals | 0.0157 | 0.0135 |
| Brain | 0.1032 | 0.0732 |
| Breasts | 0.0087 | 0.0073 |
| Gallbladder Wall | 0.0441 | 0.0484 |
| LLI Wall | 0.0145 | 0.0114 |
| Small Intestine | 0.0130 | 0.0116 |
| Stomach Wall | 0.0125 | 0.0107 |
| ULI Wall | 0.0141 | 0.0119 |
| Heart Wall | 0.0158 | 0.0137 |
| Kidneys | 0.0454 | 0.0478 |
| Liver | 0.0538 | 0.0454 |
| Lungs | 0.0116 | 0.0096 |
| Muscle | 0.0110 | 0.0093 |
| Ovaries | 0.0147 | N/A |
| Pancreas | 0.0154 | 0.0134 |
| Red Marrow | 0.0120 | 0.0098 |
| Osteogenic Cells | 0.0188 | 0.0147 |
| Skin | 0.0087 | 0.0073 |
| Spleen | 0.0122 | 0.0104 |
| Testes | N/A | 0.0090 |
| Thymus | 0.0107 | 0.0089 |
| Thyroid | 0.0101 | 0.0098 |
| Urinary Bladder Wall | 0.1368 | 0.0762 |
| Uterus | 0.0180 | N/A |
| Total Body | 0.0146 | 0.0119 |
| Effective Dose Equivalent | 0.0316 | 0.0246 |
| Effective Dose | 0.0211 | 0.0156 |

**Table S3.** Mean half-life (t_1/2_) to reach brain equilibrium for [^18^F]SDM-16 (n = 6) and [^11^C]UCB-A (n = 4) in different brain regions of nonhuman primate.

|  | *t*_1/2_ (min) | |  |
| --- | --- | --- | --- |
| Brain region | [^18^F]SDM-16 | [^11^C]UCB-A | [^11^C]UCB-A  /[^18^F]SDM-16 |
| Cingulate cortex | 143±39 | 230±132 | 1.22 |
| Frontal cortex | 139±35 | 249±97 | 1.44 |
| Insular cortex | 152±49 | 302±116 | 1.50 |
| Occipital cortex | 121±35 | 197±49 | 1.26 |
| Temporal cortex | 144±39 | 246±85 | 1.32 |
| Putamen | 117±34 | 227±82 | 1.50 |
| Caudate | 116±30 | 224±102 | 1.53 |
| Thalamus | 115±32 | 181±56 | 1.18 |
| Cerebellum | 143±39 | 137±26 | 1.18 |
| Hippocampus | 107±30 | 174±42 | 1.29 |
| Globus pallidus | 112±24 | 242±81 | 1.80 |
| Brainstem | 73±18 | 100±16 | 1.09 |
| Amygdala | 93±24 | 86±15 | 0.92 |
| Centrum semiovale | 83±18 | 166±100 | 1.98 |
| Mean ± SD | 114±25 | 197±61 | 1.73 |

***General experimental for Crystallographic Data***

Low-temperature diffraction data (ω-scans) were collected on a Rigaku MicroMax-007HF diffractometer coupled to a Dectris Pilatus3R detector with Mo Kα (λ = 0.71073 Å) for the structure of 007c-21008 and 007c-21022. The diffraction images were processed and scaled using Rigaku Oxford Diffraction software (CrysAlisPro; Rigaku OD: The Woodlands, TX, 2015). The structure was solved with SHELXT and was refined against F^2^ on all data by full-matrix least squares with SHELXL (Sheldrick, G. M. Acta Cryst. 2008, A64, 112–122). All non-hydrogen atoms were refined anisotropically. Hydrogen atoms were included in the model at geometrically calculated positions and refined using a riding model. The isotropic displacement parameters of all hydrogen atoms were fixed to 1.2 times the U value of the atoms to which they are linked (1.5 times for methyl groups). The full numbering scheme of compound 007c-21008 and 007c-21022 can be found in the full details of the X-ray structure determination (CIF), which is included as Supporting Information. CCDC number 007c-21008 and 007c-21022 contain the supplementary crystallographic data for this paper. These data can be obtained free of charge from The Cambridge Crystallographic Data Center via www.ccdc.cam.ac.uk/data_request/cif.

**007c-21008**

**Fig. S3**. A representative model with a complete numbering scheme of 007c-21008 with 50% thermal ellipsoid probability levels. The hydrogen atoms are shown as circles for clarity.

| **Table S4 Crystal data and structure refinement for 007c-21008.** | |
| --- | --- |
| Identification code | 007c-21008 |
| Empirical formula | C_10_H_9_F_2_NO |
| Formula weight | 197.18 |
| Temperature/K | 93(2) |
| Crystal system | monoclinic |
| Space group | P2_1_ |
| a/Å | 10.9097(5) |
| b/Å | 29.8863(15) |
| c/Å | 16.5828(8) |
| α/° | 90 |
| β/° | 93.905(5) |
| γ/° | 90 |
| Volume/Å^3^ | 5394.3(5) |
| Z | 24 |
| ρ_calc_g/cm^3^ | 1.457 |
| μ/mm^‑1^ | 0.123 |
| F(000) | 2448.0 |
| Crystal size/mm^3^ | 0.220 × 0.200 × 0.040 |
| Radiation | MoKα (λ = 0.71073) |
| 2Θ range for data collection/° | 5.544 to 56.558 |
| Index ranges | -14 ≤ h ≤ 14, -39 ≤ k ≤ 39, -21 ≤ l ≤ 22 |
| Reflections collected | 109695 |
| Independent reflections | 26739 [R_int_ = 0.1057, R_sigma_ = 0.0775] |
| Data/restraints/parameters | 26739/1/1513 |
| Goodness-of-fit on F^2^ | 1.021 |
| Final R indexes [I>=2σ (I)] | R_1_ = 0.0725, wR_2_ = 0.1616 |
| Final R indexes [all data] | R_1_ = 0.0958, wR_2_ = 0.1735 |
| Largest diff. peak/hole / e Å^-3^ | 0.48/-0.34 |
| Flack parameter | 0.0(3) |

**007c-21022**

**Fig. S4**. The complete numbering scheme of 007c-21022 with 50% thermal ellipsoid probability levels. The hydrogen atoms are shown as circles for clarity.

| **Table S5 Crystal data and structure refinement for 007c-21022.** | |
| --- | --- |
| Identification code | 007c-21022 |
| Empirical formula | C_10_H_9_BrFNO |
| Formula weight | 258.09 |
| Temperature/K | 93(2) |
| Crystal system | orthorhombic |
| Space group | P2_1_2_1_2_1_ |
| a/Å | 4.5206(3) |
| b/Å | 7.2219(4) |
| c/Å | 30.2962(17) |
| α/° | 90 |
| β/° | 90 |
| γ/° | 90 |
| Volume/Å^3^ | 989.09(10) |
| Z | 4 |
| ρ_calc_g/cm^3^ | 1.733 |
| μ/mm^‑1^ | 4.135 |
| F(000) | 512.0 |
| Crystal size/mm^3^ | 0.200 × 0.200 × 0.060 |
| Radiation | MoKα (λ = 0.71073) |
| 2Θ range for data collection/° | 6.25 to 54.976 |
| Index ranges | -5 ≤ h ≤ 5, -9 ≤ k ≤ 8, -39 ≤ l ≤ 39 |
| Reflections collected | 6044 |
| Independent reflections | 2194 [R_int_ = 0.0353, R_sigma_ = 0.0416] |
| Data/restraints/parameters | 2194/0/127 |
| Goodness-of-fit on F^2^ | 1.034 |
| Final R indexes [I>=2σ (I)] | R_1_ = 0.0267, wR_2_ = 0.0586 |
| Final R indexes [all data] | R_1_ = 0.0320, wR_2_ = 0.0600 |
| Largest diff. peak/hole / e Å^-3^ | 0.43/-0.32 |
| Flack parameter | 0.004(8) |
